# Shining a light on the origin of fly species

**DOI:** 10.7554/eLife.60600

**Published:** 2020-08-05

**Authors:** Hui Gong, Lucia Prieto-Godino

**Affiliations:** Neural Circuits and Evolution lab, The Francis Crick InstituteLondonUnited Kingdom

**Keywords:** olfactory, neurobiology, evolution, ecology, visual, behavior, *D. melanogaster*, Other

## Abstract

Natural light gradients within a habitat may have helped form new fly species that have differing preferences for light.

**Related research article** Keesey IW, Grabe V, Knaden M, Hansson BS. 2020. Divergent sensory investment mirrors potential speciation via niche partitioning across *Drosophila*. *eLife* **9**:e57008. doi: 10.7554/eLife.57008

New species arise when populations of the same species become so different that they no longer or rarely interbreed. Physical barriers, such as an ocean, may facilitate this process, as is the case for the different varieties of Darwin’s finches. But how do new species emerge if they coexist in the same habitat? One explanation could be a process called niche partitioning, whereby competing species use the surrounding environment in different ways, for example by feeding on different resources.

Last year, a study of 62 species of fly belonging to the *Drosophila* family, led by researchers at the Max Planck Institute of Chemical Ecology, found that the size of a fly’s antenna (the main olfactory organ) is inversely correlated to the size of its eye. i.e. species with larger eyes had smaller antennae and vice versa (Keesey et al., 2019). Both organs develop from the same structure suggesting that this inverse correlation arises through a developmental constraint. Now, in eLife, Ian Keesey, Veit Grabe, Markus Knaden and Bill Hansson – who were involved in the 2019 study – report that light variation within a forest habitat could have contributed to niche partitioning and the speciation of flies belonging to this family (Keesey et al., 2020). The team focused their study on two fly species: *Drosophila subobscura* and *Drosophila pseudoobscura.* These species are closely related and known to have large differences in the relative size of their eyes and antennae, but do not usually share the same habitat and are commonly found in Europe and North America respectively (Figure 1).

**Figure 1.**
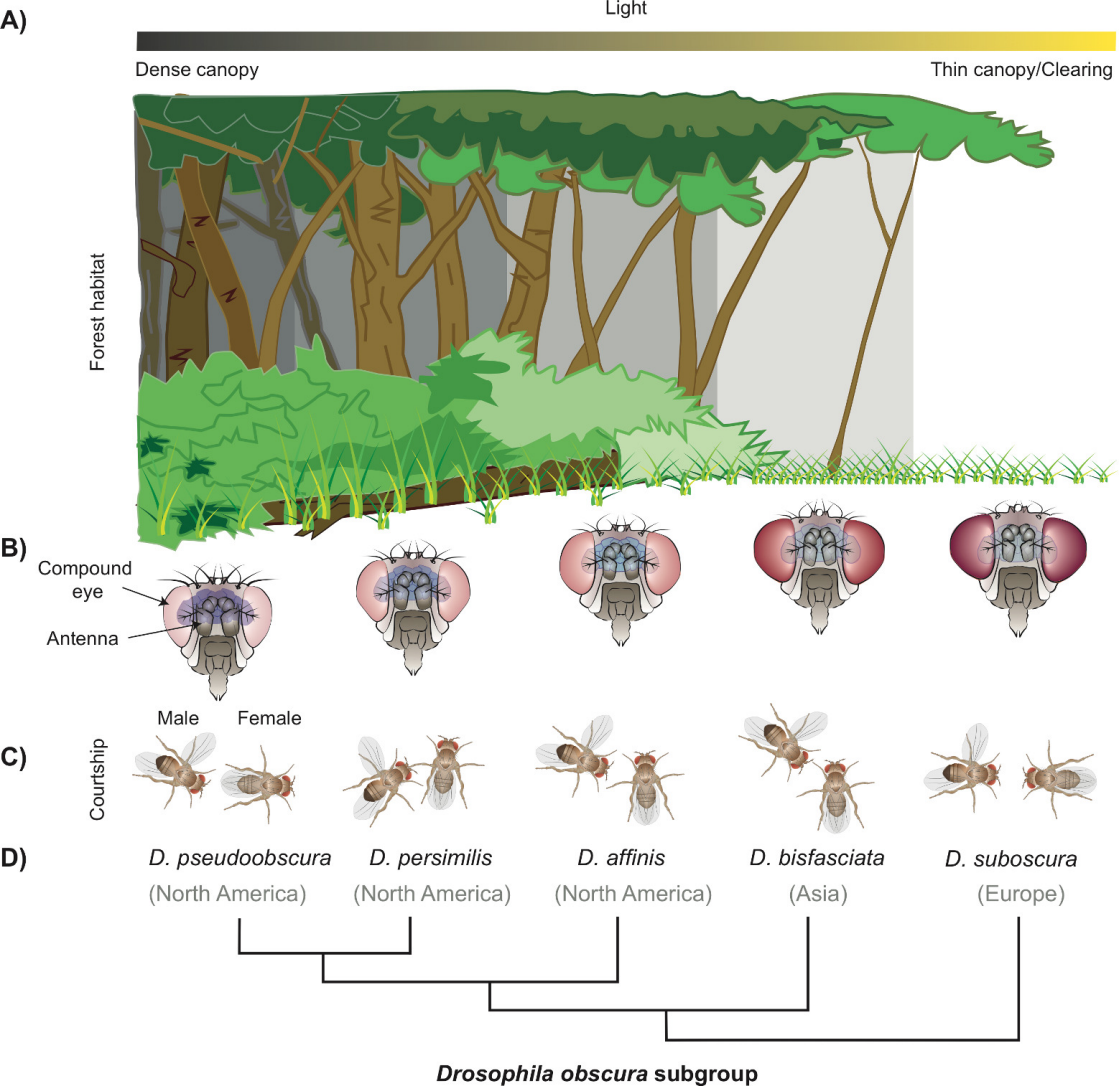
How a subgroup of flies could have become separated by niche partitioning. (**A**) The difference in density of the tree canopy covering a forest creates micro-habitats with varying levels of light, which can be a factor for niche partitioning leading to the birth of new fly species from the *Drosophila* family (**B**) Diagram showing the five fly species studied belonging to the obscura subgroup, which have an inverse relationship between the size of their eyes and antennae. *D. pseudoobscura* (left) has the smallest eyes and biggest antennae, and *D. subobscura* (right) has the biggest eyes and smallest antennae (not drawn to scale). (**C**) Diagrams illustrating the different mating rituals for each of the five species. *D. pseudoobscura* flies have the smallest eyes, are the least attracted to light, and have the least vision-dependent courtship (the male courts from the back of the female). *D. subobscura*, on the other hand, have the biggest eyes, are the most attracted to light, and have the most visually dependent courtship (fully frontal). The other species in the subgroup display a gradient of the morphology, light attraction, and mating behaviour. (**D**) Phylogenetic tree of these species and the main geographical locations where they can currently be found.

Image credit: Joe Brock.

First, Keesey et al. measured the eye size and other morphological parameters of these two species, including the number of ommatidia – repetitive units that make up the eyes of insects. Ommatidia are a bit like the pixels of a camera, in that the more flies have, the better the spatial resolution of their eyes (Gonzalez-Bellido et al., 2011; Ramaekers et al., 2019). They found that the larger eyes of *D. subobscura* reflect an increase in the number of ommatidia, rather than an increase in the size of each ommatidium, which suggests this species might have enhanced visual acuity (Figure 1B).

Keesey et al. propose that the ‘flirting’ strategy of males (i.e. their courtship rituals) may have evolved in response to these two species investing differently in the size of their eyes and antennae. *D. suboscura* males seem to rely on visual displays to attract females, for example by ‘showing-off’ their wings, whereas *D. pseudoobscura* males only approach females from the back, while singing by vibrating their wings (Figure 1C). This is consistent with previous work which showed that while *D. pseudoobscura* can mate successfully in the dark, *D. suboscura* requires light (Wallace and Dobzhansky, 1946). It is possible that the increased visual acuity of *D. suboscura* facilitated the evolution of visual courtship rituals, causing them to become sexually isolated and diverge from other species. But what other ecological factors could have driven the increased investment in the visual system?

The canopy of trees that covers the natural habitats of these two species varies greatly in density, creating distinct micro-habitats that are either dark and cool, or warm and light (Figure 1A). Further experiments showed that *D. suboscura* prefer well-lit conditions, while *D. pseudoobscura* are more likely to prefer darkness. A population of *D. suboscura* has recently colonised North America, and now share a forest habitat with *D. pseudoobscura* in some regions (Noor, 1998). It is possible that niche partitioning reduces competition between these two species, if they separate into different canopy regions.

Taken together, these findings show that visual vs. olfactory investment, dependence on vision for mating rituals, and preference for light, all vary in a correlated fashion between these two species. Yet, the order in which these features emerged is difficult to determine. One possibility is that slight differences in light preference would initially segregate flies into two micro-habitats. Flies living in better lit environments would become increasingly more visual, while flies living in the shadows might have evolved a finer sense of smell at the expense of their eyes.

Another possibility is that genetic variation within members of the same species could generate individual flies with larger eyes or antennae: these differences could lead to niche partitioning, as flies with larger eyes would be at an advantage in well-lit forest clearings, and vice versa. This would be followed by the evolution of different light preferences and mating rituals. This hypothesis is partially supported by a previous study showing that small mutations in the regulatory region of a gene called *eyeless* can change the relative size of these sensory organs within and across species (Ramaekers et al., 2019). Such simple genetic bases potentially makes the size of the eye and the antennae so easily evolvable across species.

To address the evolutionary order of these traits, Keesey et al. expanded their work to include three additional species. The results showed that *D. suboobscura* and *D. pseudoobscura* are at the two extremes in a graded variation of these three traits. One of the species examined, called *D. persimilis,* is the closest relative of *D. pseudoobscura* and shares the same habitat (Figure 1D). *D. persimilis* displayed the largest difference to its sibling species in terms of their preference for light, with smaller increases in their eye investment and visual courtship behaviour (Figure 1D). This suggests stronger evolutionary pressures for niche partitioning on light preference behaviour, with visual investment and courtship rituals further increasing this separation.

The idea that the emergence of new fly species might be due to changes in the preference for light is intriguing and inspires many more questions. For example, does this niche partitioning really occur in nature? And if so, what were the initial selection pressures favouring the differential preference for light? Could other factors correlated with canopy thickness – such as reduced risk of desiccation and irradiation – also have contributed towards this variation?

It is also unclear what neurobiological mechanism led to this initial switch in light preference. Although there is no evidence that larger eyes would make animals more attracted to light, these two traits could be linked. For example, changes in the regulatory region of *eyeless* could simultaneously affect the number of ommatidia and neuronal circuits in the eye. Given these species can be genetically manipulated (Tanaka et al., 2017), future experiments swapping their regulatory region of *eyeless* could provide some answers.

This study illustrates how studying little known fly species and their ecology can shed light on how brains evolve, and how behavioural changes can shape the evolution of new species.
